# Role of Apoptosis in Wound Healing and Apoptosis Alterations in Microgravity

**DOI:** 10.3389/fbioe.2021.679650

**Published:** 2021-06-17

**Authors:** Stefan Riwaldt, Thomas J. Corydon, Desiré Pantalone, Jayashree Sahana, Petra Wise, Markus Wehland, Marcus Krüger, Daniela Melnik, Sascha Kopp, Manfred Infanger, Daniela Grimm

**Affiliations:** ^1^Department of Microgravity and Translational Regenerative Medicine, University Clinic for Plastic, Aesthetic and Hand Surgery, Otto-von-Guericke University, Magdeburg, Germany; ^2^Department of Biomedicine, Aarhus University, Aarhus, Denmark; ^3^Department of Ophthalmology, Aarhus University Hospital, Aarhus, Denmark; ^4^Department of Experimental and Clinical Medicine, University of Florence, Florence, Italy; ^5^The Saban Research Institute, Children's Hospital Los Angeles, University of Southern California, Los Angeles, CA, United States; ^6^Research Group “Magdeburger Arbeitsgemeinschaft für Forschung unter Raumfahrt-und Schwerelosigkeitsbedingungen” (MARS), Otto-von-Guericke University, Magdeburg, Germany

**Keywords:** skin, wound healing, apoptosis, microgravity, surgery, spaceflight, space exploration

## Abstract

Functioning as the outermost self-renewing protective layer of the human organism, skin protects against a multitude of harmful biological and physical stimuli. Consisting of ectodermal, mesenchymal, and neural crest-derived cell lineages, tissue homeostasis, and signal transduction are finely tuned through the interplay of various pathways. A health problem of astronauts in space is skin deterioration. Until today, wound healing has not been considered as a severe health concern for crew members. This can change with deep space exploration missions and commercial spaceflights together with space tourism. Albeit the molecular process of wound healing is not fully elucidated yet, there have been established significant conceptual gains and new scientific methods. Apoptosis, e.g., programmed cell death, enables orchestrated development and cell removal in wounded or infected tissue. Experimental designs utilizing microgravity allow new insights into the role of apoptosis in wound healing. Furthermore, impaired wound healing in unloading conditions would depict a significant challenge in human-crewed exploration space missions. In this review, we provide an overview of alterations in the behavior of cutaneous cell lineages under microgravity in regard to the impact of apoptosis in wound healing. We discuss the current knowledge about wound healing in space and simulated microgravity with respect to apoptosis and available therapeutic strategies.

## Introduction

Programmed cell death, also known as apoptosis, is a biological process occurring in multicellular organisms (Riwaldt et al., 2017). Apoptosis is necessary to maintain the body balance and to remove cells during the organ development phase. It is involved in morphogenesis, the elimination of neoplastic cells or virus-infected cells. Furthermore, it plays a key role in myocardial infarction, cerebral ischemia, infections, neurodegenerative disorders, organ and bone marrow transplant rejection, autoimmune diseases and, among others, in cancer chemotherapy or irradiation (Schoenberger et al., 2008).

In addition, apoptosis is an important process involved in the early phases of wound healing (Greenhalgh, 1998; Wu and Chen, 2014). In normal wound healing, programmed cell death is necessary for removing inflammatory cells and afterwards for scar formation. This removal occurs without tissue damage or inflammation.

Furthermore, conditions of real and simulated microgravity can influence cell survival and increase the amount of apoptosis in different cell types and tissues (Zhao et al., 2018; Jiang et al., 2020; Mao et al., 2020; Pan et al., 2020; Sokolovskaya et al., 2020). The conquest of space is very important to us humans. In the course of the expanded exploration of space, combined with the settlement of the Moon, the installation of new Space Stations as well as planned trips to Mars and other planets, more thought must also be given to the treatment of space travelers (Patel et al., 2020). There will be an increase in crew members and space travelers on board together with more on-board and extravehicular activities. Injuries and burns are a very important focal point, an important aspect in surviving a trauma in space is the sufficient wound healing. Therefore, we need profound knowledge of the healing process and suture behavior in space. Moreover, medical evacuation times to Earth are very lengthy or not possible at all so that medical care in space has to be improved with respect to emergency surgery, treatment of acute wounds and burns on spacecrafts or space stations.

The changes of wound healing in space and the influence of microgravity on wounds need to be clarified, and the influence of apoptosis on wound healing in space is of particular interest. We will discuss the current knowledge about (1) wound healing in space; (2) alterations in healing mechanisms affected by microgravity with respect to apoptosis; and (3) possible therapeutic strategies of wound healing in space.

This review provides an overview of real and simulated microgravity research platforms. Models to study wound repair under microgravity conditions will be discussed. It summarizes the current knowledge of the impact of microgravity on the different cell types involved in wound healing with respect to programmed cell death. We will review recent findings in keratinocytes, lymphocytes, macrophages, endothelial cells, dermal fibroblasts and adipocytes. Finally, this comprehensive review reports the influence of the space environment on the biological process of apoptosis in wound healing and evaluates the new therapies to improve wound repair onboard.

## Wound Healing

Wounds are a break in the continuity of the skin, a “disruption of normal anatomic structures and functions” (Lazarus et al., 1994; Robson et al., 2001; Chhabra et al., 2017). The skin is a complex, multi-functional organ: it prevents fluid loss, stabilizes body temperature, and relays sensory inputs. In addition, it harbors a highly specialized immunological niche crucial for the maintenance of tissue homeostasis, defense, and repair (Nguyen and Soulika, 2019). Wound healing is a complex and dynamic process that is affected by wound environment and the general health and immune status of the host (Chhabra et al., 2017). Wounds can be classified in a number of different ways, depending on the type of treatment setting. For example, in a trauma setting wounds are defined based on the following factors:

### Forces Causing the Wound

Based on the different trigger factors, wounds are defined as: *blunt force wound, penetrating forces*, the latter further subdivided into *sharp forces* and *firearms force group* (Chhabra et al., 2017).

*Blunt forces* causes: Abrasion, lacerations, contusions and “lacerocontusum” wounds (combination of bruise and laceration) (Chhabra et al., 2017).

*Penetrating force causes*: incisions/cuts, stab and puncture, firearms injuries. The latter varies depending on type of projectile, muzzle velocity, distance, angle of fire and affected body area (Chhabra et al., 2017).

### Wound Depth

*Superficial*: the wound involves only the epidermis (for example, abrasions caused by frictional scraping forces) (Leaper and Harding, 2006).

*Partial-thickness*: the wound partially extends from the epidermis into the dermis (Chhabra et al., 2017).

*Full-thickness*: the wound may penetrate into the underlying tissue and involve adipose tissue, muscles, tendons, or bones. Wound healing occurs by granulation and contraction that requires more body resources and time (Chhabra et al., 2017).

*Deep wounds*: a wound that enters the main body cavities, thorax and abdomen or reaches other deep areas of the body (Oneyekwelu et al., 2017).

Another classification that is important for clinical treatment takes in account the possibility/presence of contamination.

Wounds can be also classified into surgical incisions, traumatic and accidental wounds. Surgical incisions are mainly performed and sutured in a sterile, aseptic environment, using antiseptic techniques. The use of appropriate instruments and bleeding control reduces the risk of infection. Vice versa, traumatic, accidental wounds and surgical wounds in acute care surgery can present some degree of contamination, inflammation or even infection that require specific protocols (Leaper and Harding, 2006; Oneyekwelu et al., 2017).

### Wound Healing and Clinical Practice

The different steps of tissue repair are strictly regulated by a multitude of biochemical and physical factors, including gravitational/mechanical forces acting at cellular and tissue level. Interruption, failure or alteration in one or more phases of the repair process can lead to the formation of non-healing chronic wounds or fibrotic scars. Defective healing is caused by alterations in mechanisms underlying repair, such as dysregulated immune function, chronic inflammation, impaired fibroblast function, defective ECM deposition, altered endothelial function, dysregulated apoptosis, etc (Mekhail et al., 2016).

Several factors related to patient's conditions, emergency and care procedures affect the efficiency of repair mechanisms, rate and quality of healing and the onset of wound complications: age, gender, excess weight, serious diseases (e.g., diabetes, venous or arterial diseases, infections, metabolic deficiencies, etc.), wound contamination, wound overstraining, non-physiological environment, urgency, emergency care, wound care, choice of suturing materials and techniques. Some of these factors cannot be changed, but some others can be controlled and improved in order to obtain the best therapeutic result, the healing process, and can be summarized in the following stages:

#### Stage 1: Vascular Response

Traumatized tissues induce activation of coagulation cascades leading to the formation of a fibrin mesh to fill tissue gaps (Mekhail et al., 2016). Platelets secrete several chemokines that help to stabilize the wound through clot formation and also attract and activate macrophages and fibroblasts. The latter are the protagonists of the stromal activation that leads to important downstream effects, such as epithelial cell proliferation and neoangiogenesis. This process usually lasts up to 3 d.

#### Stage 2: Inflammatory Response

Vasodilation and increased permeability of the adjacent blood vessels are present (Mekhail et al., 2016). As a result of inflammatory mediators being released by mast cells, the clinical presentation is characterized by redness, swelling, localized heat, pain, and functional limitations. This presentation might be confused with wound infection-increased capillary permeability leading to the production of exudates and essential growth factors, nutrients, and enzymes that are necessary for wound healing and that exert anti-microbial properties.

#### Stage 3: Proliferative/Granulation Phase

This stage is characterized by epithelialization, angiogenesis, granulation tissue formation, and collagen deposition (Mekhail et al., 2016). Epithelialization starts within hours after injury. Neovascularization, regulated by many factors, is needed to deliver nutrients and maintain the granulation tissue bed. Granulation tissue begins to invade the wound space about 4 days after injury. Macrophages, transforming growth factor (TGF) and tumor necrosis factor (TNF) are the promoters of angiogenesis. Collagen and other extra-cellular materials form scaffolds for the growth of new capillaries. Meanwhile, macrophages continue to supply growth factors, promoting further angiogenesis and stimulating fibroblast to produce and remodel extracellular matrix (ECM) through the synthesis of collagen, fibronectin, laminin and metalloproteases, providing strength and substance to the wound (Flanagan, 2000). The contractile activity of fibroblasts is also responsible for wound contraction and the decrease in wound size (Mekhail et al., 2016). The ability of fibroblasts to transdifferentiate in myofibroblasts strongly affects the healing evolution: myofibroblasts regulate connective tissue remodeling and wound closure by combining their own capacity of ECM biosynthesis with cytoskeletal characteristics of contractile smooth muscle cells. Finally, the process of wound re-epithelialization process takes place across the wound.

#### Stage 4: Remodeling/Maturation Phase

This final stage of wound healing may last up to 2 y from the trauma event. The scar becomes less raised and reddish, more flat, smooth and turned white due to a decreased blood supply. Mature scars have no hair, no vascularization and no sweat or sebaceous glands. Remodeling of collagen fibers to maximize tensile strength is also present (Mekhail et al., 2016).

### Wound Healing Classification

Wounds heal in three different ways: by primary intention, secondary intention, tertiary intention (Sinno and Prakash, 2013; Kumar and Reddy, 2014; Chhabra et al., 2017).

Primary intention healing occurs when the edges of the wound are closely re-approximated with minimal edema, minimal granulation tissue formation and no bacterial contamination. The wound heals in a short amount of time (usually 1 or 2 weeks), with no separation of the wound edges, and with minimal scar formation.

Healing by secondary intention occurs when there is significant tissue loss and/or bacterial contamination (Chhabra et al., 2017; Oneyekwelu et al., 2017). Wounds are usually left open to heal by granulation and contraction. Granulation tissue, rich in blood capillaries, replaces the lost tissue and wound contraction is due to myofibroblasts. This is a longer process, taking weeks or even months to complete: the proliferative phase, granulation in particular, lasts longer than in primary intention, and scars are irregular, retracting and/or hypertrophic.

Tertiary intention is the intentional delay of a wound closure (Sinno and Prakash, 2013). Wounds are left open and covered with a sterile dressing to slacken infection or inflammation. The choice of a delayed closure is intended to allow a complete cleaning of the wound and a reduction in size to close it with stitches or staples as in primary intention healing. This is a safe method of repair of contaminated, as well as dirty and infected traumatic wounds with extensive tissue loss and a high risk of contamination. These wounds are usually treated by debridement of non-viable tissue and left open until the repairing tissue gains sufficient resistance to infection to permit an uncomplicated closure (Oneyekwelu et al., 2017). Usually this takes place within 4–6 d post-injury. When closure is undertaken, skin edges and underlying tissue must be accurately and securely approximated (Kumar and Reddy, 2014; Chhabra et al., 2017; Oneyekwelu et al., 2017).

A wound can be further described by various attributes, including blood flow, oxygenation, infection, oedema, inflammation, repetitive trauma and/or insult, innervation, wound metabolism, nutrition, previous injury handling, and systemic factors (Mekhail et al., 2016). All these attributes can provide evidence of the origin, pathophysiology and condition of a wound. A major factor affecting wound healing is the general health of the host and his/her nutritional status, which can systemically affect the patient, and thereby may lead to an increased risk of infections and/or delayed recovery (Sinno and Prakash, 2013; Chhabra et al., 2017).

In conclusion, wounds remain an ongoing challenge, due to early and late complications that may possibly arise and develop toward an increase in morbidity and mortality. Considering the dynamic evolution of the healing process, this shall require a constant, systematic and consistent evaluation with continuous reassessment of their extent, type and severity.

## Apoptosis

Programmed cell death or apoptosis is a biological process responsible for normal cell turnover in various organs. An example is the apoptosis of lymphocytes in the tonsil. In case of a chronic tonsillitis, the relationship between apoptosis and proliferation of lymphocytes in tonsillar follicles can be disturbed (Avramović et al., 2015). Inappropriate apoptosis is a key factor in various diseases including cancer, ischemia of the heart and brain, autoimmune or neurodegenerative diseases (Elmore, 2007; Schoenberger et al., 2008). In addition, programmed cell death has important functions on growth, differentiation, morphogenesis, organ development and tissue homeostasis. This type of cell death is able to modulate life and death of cells and exhibits a known therapeutic potential in cancer (Schoenberger et al., 2008; Grimm et al., 2011).

Apoptosis shows characteristic morphological cellular changes such as cell shrinkage, chromatin condensation, membrane blebbing and apoptotic bodies without inflammation (Kerr et al., 1972; Böhm and Schild, 2003; Kossmehl et al., 2003). The morphological changes of programmed cell death were first described by Kerr et al. (1972). These changes occur as follows: The first step is cell shrinkage, followed by a stop of adhesion to the neighboring cells. The cell membranes and the organelles of the apoptotic cells remain intact. This step is followed by chromatin condensation, which is also called pyknosis. Membrane blebbing on the cellular membrane occurs and the nucleus ruptures into fragments, a process called karyorrhexis (Kossmehl et al., 2003). Cytochrome c is released after rupture of the mitochondrial membrane, which activates a cascade of degradation reactions resulting in the secretion of lysosomal enzymes. The result of the enzymatic activity is the formation of small cell particles completely enclosed by cell membranes containing cell fragments. These particles are called apoptotic bodies. The cellular fragments are cleared quickly by phagocytosis without an inflammatory reaction (Taylor et al., 2008).

Apoptosis comprises a complex mechanism with specifically interacting pro-apoptotic and anti-apoptotic factors. It can be initiated by external signals via death receptors. This very well-defined pathway is called extrinsic pathway of apoptosis (EPA). These death receptors comprise FAS/CD95 and the receptors for tumor necrosis factors (TNF-R) (Ashkenazi and Dixit, 1998). The ligand-receptor binding results in the initiation of the cell death machinery. The canonical pathway of the EPA starts with the binding of the tumor necrosis factor receptor superfamily (TNFRSF) members to cognate trimeric ligands of the tumor necrosis factor superfamily (TNFSF). These ligands are either soluble or expressed on the cell surface of another cell.

FASL binding to FAS results in the trimerization of FAS. This process is followed by recruiting the initiator caspase-8 via the FAS-associated death domain protein (FADD). Then the plasma membrane-associated death-inducing signaling complex (DISC) converts procaspase-8 into the active form of caspase-8 (Yang, 2015; Siegmund et al., 2017). The next step is the activation of the effector caspase-3 (Siegmund et al., 2017). Caspase-3 is a cysteine–aspartic acid protease, which is known to cleave various targets and to initiate cell death.

Activated caspase-8 induces apoptosis first by the activation of caspase-3 and second by cleavage of BH3 interacting-domain death agonist (BID), a pro-apoptotic Bcl-2 family protein. The cleaved BID now termed truncated Bid (tBID) translocates to the mitochondria. Afterwards it induces the release of cytochrome c, which is a key protein that initiates the intrinsic pathway of apoptosis (IPA). Cytochrome c is normally localized in the compartment between the inner and outer mitochondrial membranes and its release results in the activation of caspase-9 and−3 (Taylor et al., 2008). The apoptotic protease activating factor 1 (Apaf-1) is involved in the cytochrome-c-dependent activation of caspase-3 (IPA). Mitochondrial cytochrome c is released into the cytosol and binds to Apaf-1 and thus forms the apoptosome. Apaf-1 subsequently activates the caspase cascade. Afterwards, the apoptosome recruits and activates the inactive pro-caspase-9. Active caspase-9 is an initiator caspase and activates effector caspases, which results in the activation of several steps leading to apoptosis.

Cysteine aspartases (caspases) are enzymes involved in the process of apoptosis. EPA and IPA merge at the activation of a procaspase. Apaf-1 activates procaspase-9, while Fas-associated protein with death domain (FADD) activates procaspase-8. The caspases-2,−8,−9, and−10 initiate and activate other caspases, while the caspases-3,−6, and−7 execute the process and cleave proteins at the site of an aspartate. Intracellular type I keratin and other intermediate filament destruction by these enzymes results in the characteristic morphological signs of apoptosis (Oshima, 2002).

NF-κB (nuclear factor κ-light-chain-enhancer of activated B cells) is a protein complex involved in DNA transcription, cell survival and cytokine production (Taniguchi and Karin, 2018). NF-κB exhibits various transcriptional regulatory functions and is a key player in apoptosis (Ghobrial et al., 2005) and it regulates the immune response to infections (Hayden et al., 2006). Incorrect regulation of NF-κB is among others associated with cancer and infections (Siebenlist et al., 1994), which might be important for wound healing processes in space. NF-κB is inactivated by binding to IκB (inhibitor of NF-κB). Moreover, the IκB degradation induces the translocation of NF-κB into the nucleus, where it changes transcription (Song et al., 2012) and triggers various processes like survival or detachment and aggregation of cells to three-dimensional aggregates (Kopp et al., 2018).

The transcription factor p65 (nuclear factor NF-κB p65 subunit) is a protein which is encoded by the *RELA* gene. The presence of the transcription factor p65 in the skin (epidermis) inhibits epithelial cell death induced by Fas ligand, tumor necrosis factor-κ, and microbes (Seitz et al., 2000).

The inhibitor of apoptosis protein (IAP) family regulates the activity of caspases, survival, and proliferation by binding to the baculovirus IAP repeat (BIR) domains (Cossu et al., 2019). Moreover, IAPs also control various pathways. The levels of X-linked IAP and other IAPs are often associated with progressive cancer and correlate with prognosis (Cossu et al., 2019). Therefore, compounds targeting the IAPs are of interest in cancer therapy (Saleem, 2013).

Today most of the key players in cellular apoptosis regulation are identified and can be targeted by therapeutic strategies. Examples are drugs targeting the EPA (FAS, tumor necrosis factor-α and tumor necrosis factor related apoptosis-inducing ligand), or compounds binding to factors of the IPA (Bcl-2 family) or Poly (ADP-ribose) polymerase (PARP) inhibitors.

## Autophagy

Autophagy is a physiological process, which serves to degrade misfolded or damaged proteins or dysfunctional organelles such as the endoplasmic reticulum or mitochondria in a catabolic fashion and subsequently reuse some of the degradation products (Mizushima, 2007; Glick et al., 2010). Besides its function in cell maintenance, differentiation and survival, autophagy has also been implicated in the development of diseases (reviewed in Levine and Kroemer, 2008). Interestingly, it has also been shown, that elevated autophagy activity in the skin can lead to impaired cutaneous wound healing (Guo et al., 2016).

Only few studies so far have investigated the influence of microgravity on autophagy. Osteoclasts and endothelial cells are the most widely used cells types. When preosteoclasts were exposed to simulated microgravity on an RCCS for 24 h, the cells showed an elevated autophagy activity, which was linked to an increased syncytin-A expression (Ethiraj et al., 2018) and increased differentiation into osteoclasts (Sambandam et al., 2014) reported similar findings. The process of simulated microgravity-induced formation of osteoclasts could be suppressed by melatonin (Yoo et al., 2016) as well as 4-acetylantroquinonol B (Wu et al., 2020) by inhibiting the autophagy pathway. It can be speculated that counteracting autophagy in microgravity might be a suitable countermeasure for bone loss in space.

Several studies conducted on vascular endothelial cells reported an upregulation of autophagy under simulated microgravity. In contrast to the findings in osteoclasts, however, the results hinted toward a protective effect of autophagy against microgravity-induced stress (Wang et al., 2013; Li et al., 2018, 2019). Recently, it was also found that after 10 d of simulated microgravity on a rotating wall vessel, a disorganization of the actin cytoskeleton triggered autophagy/mitophagy and led to a reduction of mitochondrial content, oxygen consumption, and maximal respiratory capacity in human primary endothelial cells (Locatelli et al., 2020).

Similarly, it could be demonstrated, that simulated microgravity induces autophagy in TCam-2 seminoma cells (Ferranti et al., 2014) as well as human Hodgkin's lymphoma cells (Jeong et al., 2018), pointing toward a possible new target for anti-cancer therapy.

## Platforms for Experiments in Real Microgravity

**Figure 1:**
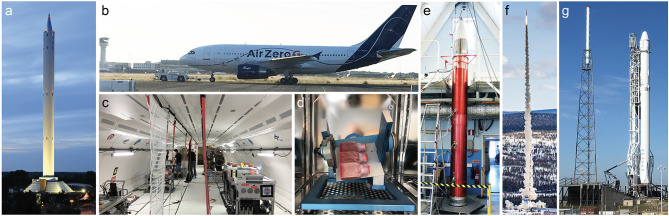
Images of platforms for research experiments in real and simulated microgravity conditions. **(a)** ZARM Drop Tower, Bremen Germany. Ground-based facility providing installation of the experimental set up in an airtight capsule which is released in a tube inside the tower. High payload masses and up to 10 s of real microgravity can be provided (credit ZARM Drop Tower Operation and Service Company). **(b)** Airbus A310 AirZeroG parabolic aircraft operated by Novespace, Bordeaux, France. Repeated periods of ~22 s of real microgravity can be obtained during parabolic flight maneuvers. **(c)** Experimental area inside the AirZeroG aircraft with different experiment racks. An enormous advantage of parabolic flights is that experimenters have the opportunity to access hardware during microgravity exposure. **(d)** Desktop Random Positioning Machine invented and constructed by Airbus, Defense, and Space, Leiden, NL. This ground-based instrument accommodates large sample sizes and the samples are rotated around two axes in order generated multidirectional *g*-force thereby canceling the cumulative gravity vector at the center of the device. **(e)** Payload of a TEXUS-type sounding rocket (SSC, ESRANGE, Kiruna, Sweden). **(f)** Launch of a TEXUS-51 sounding rocket from SSC, ESRANGE, Kiruna, Sweden, which empowers microgravity for ~6 min. **(g)** SpaceX CRS-8 rocket on the launch pad, Kennedy Space Center (KFC), FL, USA providing resupply and experiments to ISS. Currently, the ISS is the only option that provides long-time exposure to microgravity (months or longer).

The term microgravity in general refers to the still existing residual accelerations. When gravitation is the only force acting on an object then the object is in free fall, and hence, it will experience microgravity. As outlined in Figure 1, there are several opportunities to expose objects to real microgravity. Most importantly, these differ in the duration of microgravity (seconds to months), but several other parameters, like quality of microgravity (10^−2^-10^−6^ *g*), availability, experiment series (daily to 1 per several years), preparation time (month to years), cost, hardware accessibility (hours to months), masses of payload, and degree of automatization change dramatically between the platforms (Sabbatini, 2014; Hemmersbach et al., 2018; Amselem, 2019; Prasad et al., 2020a,b). On this basis, the available microgravity platforms will be described briefly in this chapter.

The most common way to conduct experiments in real microgravity is to use drop towers (or shaft towers or drop wells), which are ground-based research facilities. The duration of the microgravity time provided by operational drop towers is relatively short—3.5–4.7 s depending on the height of the tower and technical facilities. In the case of the 146 m tall ZARM drop tower in Bremen, Germany (see Figure 1a), the experimental time can be extended to 9.3 s due to a clever catapult system. The biggest microgravity drop well facility in the world, JAMIC, is 710 m deep (underground) and located in Japan. It can provide microgravity for the duration of 10 s, although it is currently not operational. The NASA Glenn shaft and Beijing drop tower provide 5.2 s and 3.6 s of microgravity, respectively (Zhang, 2005; Könemann et al., 2015). The experimental setup is installed into an airtight capsule which is released in a tube within the tower. To eliminate the effect of friction forces and drag most drop tower experiments are performed in an air free (vacuum) tube. After the free fall the capsule is exposed to substantial deceleration phases during the dampened impact. To double the microgravity time the capsule can be catapulted upward from the bottom of the tube, by which the experiment is exposed to a high acceleration event, followed by a free fall. Among the available drop towers, the facility at ZARM provides the best microgravity conditions of ~10^−6^ *g* (Selig et al., 2010; Corydon et al., 2016). Drop towers provide several advantages including low-cost access to research in microgravity conditions, high flexibility, high payload masses (up to 265 kg), good availability, hardware accessibility a few hours before and after drop, short experiment planning phase, fast turn-around time, they allow the execution of a series of experiments within a few days with direct intervention by research teams to introduce modifications between drops, and vibrations during microgravity are very low. As a consequence of the short microgravity time and the setup of drop towers, experiment packages must work fully autonomously.

Parabolic flights can be considered as another stepping stone to space (Sabbatini, 2014). Parabolic flights are conducted in a customized aircraft and provide 22 s of microgravity time (Figures 1b,c). To produce a free fall period (microgravity) the aircraft is flying parabolic trajectories consisting of three phases: From steady flight, the aircraft climbs at a gradually steeper angle until 50° is reached. In this first phase, the experiments as well as the personal onboard the aircraft will experience hypergravity in the range of 1.8–2 *g* (Ma et al., 2014; Pletser et al., 2016). Next, the pilot reduces the thrust to compensate for drag and the aircraft will follow a ballistic trajectory, also known as a parabola. In this second phase, the aircraft is in free fall and the experiments will experience microgravity in the range of ~10^−2^ *g* (Corydon et al., 2016). At the apex of the parabola, the aircraft nosedives until a decline angle of ~42° is reached, thereby entering the third and last phase of the parabola, in which the airplane again experiences hypergravity (1.8–2 *g*). Finally, the pilot levels out the aircraft for steady flight. The free fall phase persists for 22 s, whereas the two hypergravity phases before and after the free fall phase each last for 20 s. A parabolic flight campaign provided by Novespace, Bordeaux-Merignac, France, has a duration of 3–4 d with 31 parabolas on each flight day. A huge benefit of parabolic flights is that the scientists have the opportunity to be onboard the aircraft during flights, thereby allowing them to direct hardware access during microgravity exposure. Hence, full automatization is not required. In addition, experiments involving microgravity effects on human physiology can be performed. The availability is good, half a dozen flight campaigns are annually conducted by DLR, CNES and ESA in Europe. Typically, the hardware is handed over for installation in the aircraft a few days before the first flight day, and experiments are transferred as soon as a few hours before take-off. High payload masses up to 200 kg can be accommodated by parabolic flights and only few months of experiment planning are needed. The consecutive flight days allow conduction of a series of experiments with modifications between the flights. Hypergravity and vibrations are unavoidable events of parabolic flights, which need to be taken into consideration when evaluating the impact of microgravity (Corydon et al., 2016).

A longer microgravity-exposure demands a visit to space, and suborbital spaceflights in terms of sounding rockets can be an option. For the TEXUS-type sounding rocket (DLR) the payload is in free fall for a period of 6 min (Figures 1e,f), and time spans as long as 14 min of microgravity-exposure are delivered by the MAXUS platform (ESA/DLR) (Sabbatini, 2014). In both cases microgravity below 10^−4^ *g* is obtained. The sounding rockets follow a parabolic trajectory with an apex of ~250 km (TEXUS) or 750 km (MAXUS) above the Earth's surface. The hardware is typically installed in the rocket a few days before lift-off, whereas the experiments (e.g., late access modules) are handed over 1–2 h prior to launch. Inherently, experiment packages must be fully autonomous. The payload will be subjected to hypergravity and vibrations during lift-off and landing which need to be taken into consideration (Corydon et al., 2016). Otherwise, residual accelerations are very low. Recovery of the payload after landing takes a few hours, bad weather conditions might double or triple this period. Operating costs are considerably higher compared to the previously discussed platforms.

Building a road to space is not only an endeavor for public institutions but has also, step by step, become a strategy for private companies. Blue Origin and Virgin Galactic are noticeable examples of suborbital spaceflight options delivering up to 4–6 min of microgravity (Vanderploeg et al., 2011). Key to these companies is to make access to space less expensive and more reliable through reusable launch vehicles, including development of the so-called VTVL technology (vertical take-off and vertical landing). All experiments must be fully autonomous.

Exposure that lasts up to 5 days can be obtained onboard a spacecraft during a resupply mission to the International Space Station (ISS). However, only a few studies have been reported using this rare option (Horn et al., 2007). An unmanned spacecraft can increase the duration of microgravity time. These are usually satellites, equipped with return vehicles designed to descent back to Earth (e.g., Russian Bion-M- and Foton-M 4-type or Chinese FSW-type) which can provide exposure that lasts a few weeks. These options are likewise very rare: The first Bion-M and Foton-M4 missions were launched in 2013 and 2014, respectively, and relaunches are scheduled in 2023. Experiment packages must be fully autonomous, and years of preparation time should be anticipated. Payload masses are typically low.

As China became the third country sending humans to space in 2003, scientific experiments were conducted on board the Shenzhou spaceships also. Shenzhou missions are launched on the Long March rocket 2F from the Jiuquan Satellite Launch Center.

In order to gain long-term access to real microgravity, we took advantage of the opportunity to participate in the Shenzhou-8 Spaceflight mission, accomplished by a joint endeavor of the German Space Agency and the Chinese Manned Space Engineering Office (CMSEO). Shenzhou-8 was launched by means of a Long March rocket 2F in the Cosmodrome Jiuquan on November 1, 2011 (Pietsch et al., 2013). SIMBOX (Science in Microgravity BOX) contained 17 experiments (https://www.dlr.de/rd/en/desktopdefault.aspx/tabid-2283/3420_read-32536/). Currently, manned and unmanned Chinese spacecrafts are available for scientific endeavors.

The ISS is currently the only option that provides exposure to microgravity for months or longer. It is however, also the most expensive flight option. On the other hand, there are many flight opportunities (e.g., NASA/ESA missions operated by SpaceX or Russian Soyuz missions, Figure 1g). A microgravity quality of 10^−4^ *g* is obtained. In general, experiment packages must be fully autonomous as crew members have a tight schedule. The hardware is typically handed over 1–2 d before lift-off. Experiments should be designed in a way that allows for a 3–5 day delay of the experiment start due to the flight time to the ISS. Accelerations are low during lift-off and the flight. Access to experimental data is possible during or after the experiment has been completed. Hand-over of the hardware is possible several days after return to the Earth.

Moreover, China is presently constructing a Chinese Space Station (CSS) named Tiangong, which will be approximately one-fifth the mass of the ISS. The CSS should consist of multiple modules allowing scientific experiments to be carry out onboard, and it is anticipated to be operational by 2022.

## Devices to Study Altered Gravity Conditions on Earth

Access to space is expensive, limited and a logistic challenge. On top of this, repetitions of the experiments as well as the number of samples are severely limited (Brungs et al., 2016). To simulate microgravity on Earth a variety of ground-based alternatives have therefore been developed (Herranz et al., 2013; Brungs et al., 2016). One of the simplest devices used for small samples, like cells, is the rotating wall vessel (RWV). In this device simulated microgravity is obtained by placing the cells in a horizontal vessel rotating with a speed that cancels sedimentation (Schwarz et al., 1992). Ideally, the RWV device produces a low shear fluid environment that is optimized for tissue growth and suspension cultures (Klaus, 2001).

In an attempt to reproduce the quiescent, unstirred fluid conditions achievable in orbit, clinostats have been developed for cell culture systems. The so-called clinorotation provided by the device also seeks to simulate microgravity by canceling sedimentation. In clinostats this is accomplished by subjecting the cells to rotations, either constantly or through directional alterations. In the former case the classical two-dimensional (2D)-clinostat rotates a sample perpendicular to the gravity vector (Hemmersbach et al., 2006; Herranz et al., 2013). If samples do not exceed a radius of 1.5 mm around the rotation axis, and the speed of rotation is limited to <60 rpm, a theoretical *g*-force in the range of 10^−2^-10^−3^ *g* can be obtained (Hemmersbach et al., 2006). Even though the sample size of the 2D-clinostat platform is limited, shear forces and fluid disturbances are low (Hemmersbach et al., 2006). The 3D-clinostat uses a comparable principle but can add another dimension. Hence, the sample rotates around two axes in order to provide a status of “vector-averaged gravity,” and the generated multidirectional *g*-force thereby cancels the cumulative gravity vector at the center of the device (Hoson et al., 1997; Schwarzenberg et al., 1999). The quality of *g*-force provided by a 2D-clinostat is comparable to that of 3D-clinostats. In the latter case, randomized directional changes are delivered by a random positioning machine (RPM) (Figure 1d). During operation, the movement of the sample describes a sphere, and studies have demonstrated that at 70 mm around the rotation point the *g*-force is in the range of 10^−3^-10^−4^ *g* (Van Loon, 2007). Compared to the 2D clinostat the RPM clearly provides a larger sample size. However, shear forces arise, especially on the edges of the culture flasks, which need to be taken into consideration (Wuest et al., 2015, 2017).

For larger samples, e.g., mice or rats, the rodent hindlimb suspension (HS) model has been used to study various aspects of musculoskeletal loading (Simske et al., 1994; Morey-Holton and Globus, 2002). In this model the hindlimbs are elevated, unloading them from any force, to produce a 30 degrees head-down tilt. Besides simulating reduced forces on the skeleton and muscles of the hindquarters the model also results in a cephalad fluid shift (Morey-Holton and Globus, 2002). The typical duration of a HS experiments is in the range of days or a few weeks.

To simulate microgravity effects in humans the head-down bed rest (HDBR) model can be applied. In this model the subjects adhered to a strict 6° head-down tilt bed rest, and several studies have demonstrated that it is replicating the effects of spaceflights (Pavy-Le Traon et al., 2007). The HDBR model induces several physiological changes, including a cephalad fluid shift, promotes movement of abdominal organs toward the chest, reduces the hydrostatic gradient in the cardiovascular system, and results in reduced loading on the musculoskeletal system (Fortney et al., 2011). Notably, in all cases it is important to compare the results from experiments conducted under simulated microgravity with data obtained from experiments performed under real microgravity.

## Impact of Microgravity on Apoptosis in Different Dermal Cell Types

**Figure 2:**
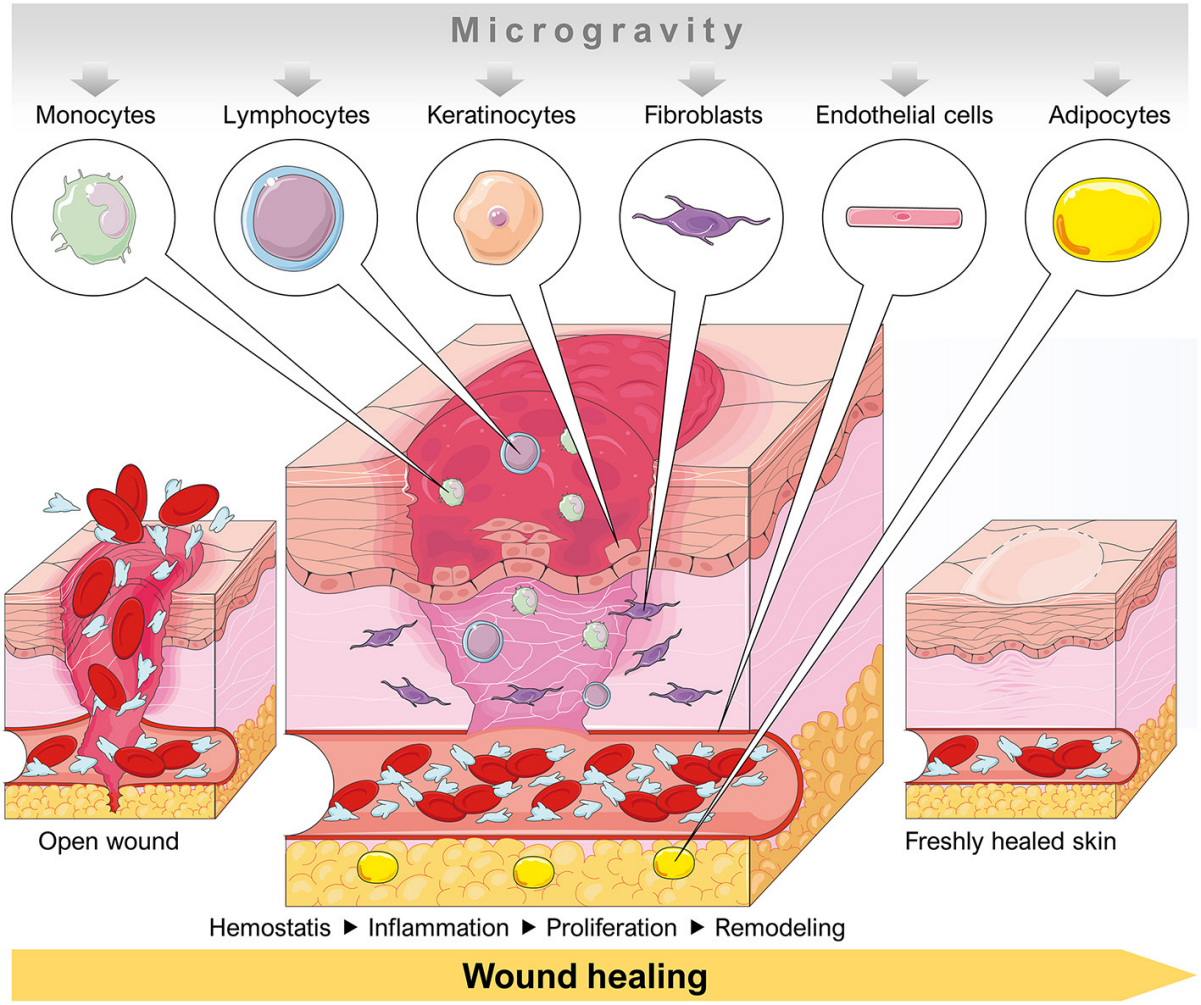
Schematic overview of a dermal wound healing process involving cell types that are influenced by a microgravity environment. Parts of the figure are drawn using pictures from Servier Medical Art (https://smart.servier.com), licensed under a Creative Commons Attribution 3.0 Unported License (https://creativecommons.org/licenses/by/3.0).

Several dermal cell types had been investigated in space and under conditions of simulated microgravity. Various studies focused on the influence of microgravity on programmed cell death (Figure 2). A detailed summary of these publications in respect to apoptosis detection in different dermal cell types is given in Table 1.

**Table 1:** Summary of selected articles addressing research on primary cells and specialized differentiated cells *in vitro* cultured under real or simulated microgravity, ordered by cell type.

**Cell line**	**Cell type**	**Device and exposure duration**	**Findings in microgravity (μ*g*)**	**References**
HaCat	Keratinocytes, human	RPM (6, 24, 60 h); 1 *g*-samples	Triggers EMT	Ranieri et al., 2017
HEK001	Epidermal keratinocytes, human	HARV (3, 4, 4 d + recovery, 9 d + recovery, 10 d + recovery)	Gene expression profiling, reduced cell death	Clement et al., 2008
–	Epidermal keratinocytes, rats	Space Shuttle Columbia during the STS-58 mission (SLS-2)	Accumulation of cellular p53	Ohnishi et al., 1996
Primary lymphocytes	Lymphocytes (PBLs), human	RWV (24, 48, 72 h); 1 *g*-samples	Increased frequency of apoptosis and decreased cell proliferation	Girardi et al., 2014
Jurkat T	Lymphocytes	Space Shuttle flight STS-80 (Columbia) and STS-95 (Discovery) (75 h); 1 *g*-samples	Increased rate of apoptosis	Lewis et al., 1998
Primary lymphocytes	Lymphocytes (PBMCs), human	RPM (72 h); 1 *g*-samples	Increased apoptosis Calcium-dependent 5-LOX activation	Maccarrone et al., 2003
Primary lymphocytes	Lymphocytes (PBMCs), human	ISS (2 d); 1 *g*-samples	Increased rate of apoptosis Increased DNA fragmentation and 5-LOX activity	Battista et al., 2012
Primary lymphocytes	Lymphocytes (PBMCs), human	RCCS (18–24 h); 1 *g*-samples	Reduced apoptotic cell death	Risin and Pellis, 2001
Lymphocytes	Human B-lymphocytes Human T-lymphocytes	Clinostat (4, 72 h, 7 d); 1 *g*-samples	Decreased DNA repair capacity Reduced expression of cell-cycle genes Downregulation of pro-apoptotic genes	Kumari et al., 2009
U937	Macrophage, human	RWV (24, 72 h); 1 *g*-samples	Reduce cell growth, no sign of apoptosis induction	Maier, 2006
U937	Macrophage, human	2D clinostat; 1 *g*-samples	Regulation of ICAM-1	Paulsen et al., 2015
U937	Macrophage, human	Space Shuttle Atlantis during the STS-81 mission	Modified translocation of protein kinase C isoform	Hatton et al., 2002
NR8383	Macrophages, rat	Spaceflight to the ISS (up to 500 min); 1 *g*-samples	Rapid adaptation to reduced gravity	Thiel et al., 2017
NR8383	Macrophages, rat	2D PMT-clinostat, parabolic flight (22 s); and 1 *g*-samples	ROS production in macrophages is a gravisensitive process	Adrian et al., 2013; Brungs et al., 2015
Differentiated HPCs (Lin^−^)	Macrophage, mouse	Tianzhou-1 cargo spaceflight, SJ-10 satellite (12 d), and RCCS (12 d)	Suppressed macrophage development	Shi et al., 2020
Primary macrophages	Macrophage, mouse	RCCS (28 h); 1 *g*-samples	Tumor necrosis factor-related apoptosis	Wang et al., 2014
RAW264.7	Macrophage, mouse	RCCS (28 h); 1 *g*-samples	Tumor necrosis factor-related apoptosis	Wang et al., 2014
EA.hy926	Endothelial cells, human	3D clinostat (4, 12, 24, 48 and 72 h), VEGF (10 ng/ml), 1 *g*-samples	Caspase-3, Bax, Fas, and 85-kDa apoptosis-related cleavage fragments increased Anti-apoptotic effects by VEGF	Infanger et al., 2006
EA.hy926	Endothelial cells, human	3D clinostat (up to 10 d), 1 *g* samples	Caspase-3, Bax, and Bcl-2 protein content elevated	Infanger et al., 2007
PAEC	Porcine aortic endothelial cells (PAEC) overexpressing VEGFR2	RPM (72 h), 1 *g* samples	Proapoptotic signals increased, Anti-apoptotic and proliferation/survival genes were down-regulated	Morbidelli et al., 2005
HPMECs	Human pulmonary microvascular endothelial cells	Clinostat (72 h), 1 *g* samples	TUNEL: elevated apoptosis in Clinostat-exposed cells Increased expression of NF-κB	Kang et al., 2011
HMEC-1	Endothelial cells, human	Ground experiment, 1 *g*-samples	Determination of the biological and engineering requirements that will allow retrieval of suitable samples after culturing, fixing and storing ECs in space	Balsamo et al., 2014
HPMECs	Human pulmonary microvascular endothelial cells	Clinostat (72 h); 1 *g*-samples	miR-503-5p induced apoptosis and decreased Bcl-2	Tang et al., 2019
HUVEC	Human umbilical vein endothelial cells	RWV (48 h)	miR-27b-5p could protect vascular endothelial cells from apoptosis partially via regulating the expression of ZHX1	Pan et al., 2020
HUVEC	Human umbilical vein endothelial cells	2D-Clinostat	Apoptosis, pro-inflammatory cytokine production, nuclear factor kappa B (NF-κB)/IκB signaling	Jiang et al., 2020
CVEC	Choroidal vascular endothelial cells, human	RCCS (24 h, 72 h), 1 *g* samples	Activated Bcl-2 apoptosis pathway and PI3K/AKT pathway Elevated Bax, Caspase3, and Cytochrome C	Zhao et al., 2021
HUVEC	Human umbilical vein endothelial cells	RWV (4 d, 10 d)	HSP70 up-regulation as adaptive response to RWV exposure	Cazzaniga et al., 2019
CF	Cardiac fibroblasts, porcine	RPM (24 h) bFGF, VEGF, 1 *g* samples	Increase in apoptosis in RPM samples, VEGF and bFGF reduced the amount of apoptosis	Ulbrich et al., 2010
WI-38	Quiescent normal human fibroblasts, derived from fetal lung	Space Shuttle, STS-93 mission (5 day spaceflight); ground controls	Changes in gene expression associated with cellular stress signaling, directing cells to either apoptotic death or premature senescence	Liu and Wang, 2008
STO	Mouse fetal fibroblast cells	RPM, 24 h, 1 *g* controls, irradiation (0, 0.5, 1, or 4 Gy)	Decrease in apoptosis at all doses as measured by caspase-3 activity	Beck et al., 2012
NIH3T3	Fibroblasts, NIH Swiss mouse embryo	RPM, 72 h, 1 *g* samples	Reduction in cell number	Cialdai et al., 2020
Primary cells	Adipocytes (ADSCs), human	Clinostat, 1, 3, 7 d; 1 *g*-samples	Altered gene expression of ECM and adhesion molecules, which potential may facilitate wound healing	Ebnerasuly et al., 2018

*2D, two-dimensional; 3D, three-dimensional; ADSCs, adipose-derived stem cells; CF, cardiac fibroblast; CVECs, choroidal vascular endothelial cells; EMT, epithelial-mesenchymal transition; HARV, high aspect ratio vessel; HMEC, human microvascular endothelial cell; HPC, Hematopoietic progenitor cell; HPMEC, human pulmonary microvascular endothelial cell; HUVEC, human umbilical vein endothelial cell; ISS, International Space Station; NIH, National Institutes of Health; PAEC, porcine aortic endothelial cell; PBL, Peripheral blood lymphocyte; PBMC, peripheral blood mononuclear cell; PMT, photomultiplier; RCCS, rotary cell culture system; ROS, reactive oxygen species; RPM, random positioning machine; RWV, rotating wall vessel; r-μg, real microgravity; s-μg, simulated microgravity; SLS, space launch system; STS, space transportation system. VEGFR2, vascular endothelial growth factor receptor 2*.

### Keratinocytes

Keratinocytes (KC) are the most abundant and specialized epithelial cell type in the epidermis and apoptosis of KC presents a vital pattern in the modulation of proliferation and maintenance of epidermal thickness and removal of premalignant cells. The data regarding KC apoptosis under simulated and real microgravity is very limited. Ranieri et al. demonstrated that apoptosis of the keratinocytes is not affected by the exposure to the simulated microgravity (Ranieri et al., 2017). In another study Clement et al. stated that ground-based simulated microgravity conditions, by using high aspect ratio vessel (HARV) bioreactors, results in decreased cell death of 3 and 4 day basal-like type of immortalized human epidermal keratinocytes (Clement et al., 2008). Earlier on, Ohnishi et al. reported a 4-fold increase in the content of p53 in skin keratinocyte cells of rats that were taken into space on SLS-2 (Columbia, STS-58) for 14 d, which could protect the damaged cell allowing for recovery and resumption of cell proliferation and reduced apoptosis process (Ohnishi et al., 1996). Despite the limited knowledge the obtained findings suggest that real and simulated microgravity influence the apoptosis process in keratinocytes.

### Lymphocytes

Apoptosis plays a vital role in maintaining epidermal structure and homeostasis in the skin. Keratinocytes are the major cell type in the epidermis. Apoptotic cell death is critical for balancing of keratinocyte proliferation as well as for formation of the stratum corneum (Raj et al., 2006). Apoptosis of keratinocytes occurs not only during normal keratinization but also in response to various intracellular or extracellular stimuli such as genetic defects, UV radiation, exposure to real and simulated microgravity. The keratinocytes act as the first line of innate immune defense against infection through direct activation of primed T lymphocytes and NK cells through major histocompatibility complex I (MHC-I). Adaptive immunity requires the production of specific T lymphocytes to identify an antigen with precision help of B cells to produce specific antibodies that bind to the microbes in a “lock-and-key” fashion.

There is evidence that exposure of lymphocytes to spaceflight as well as to simulated altered gravity conditions created by microgravity-simulating devices results in elevated apoptosis of immune cells (Prasad et al., 2020a). Lymphocytes cultured in simulated microgravity conditions exhibited elevated apoptosis and reduced cell proliferation (Girardi et al., 2014). Previous studies revealed that the rate of apoptosis in Jurkat T lymphocytes was increased in microgravity conditions (Lewis et al., 1998; Cubano and Lewis, 2000, Battista et al., 2012), which was reflected in time-dependent release of apoptosis-related factors like Fas/APO1 in the culture medium during exposure of 2 d real microgravity aboard different space shuttle flights conditions (Lewis et al., 1998, Cubano and Lewis, 2000). Furthermore, microgravity led to increased DNA fragmentation, poly (ADP-ribose) polymerase (PARP) protein expression and p53 and calpain mRNA. These changes were paralleled by an early increase of 5-lipoxygenase (5-LOX) activity (Battista et al., 2012). During the 28^th^ Parabolic Flight Campaign, the European Space Agency has demonstrated that microgravity directly enhances catalytic efficiency of pure lipoxygenase, up to ~4-fold over the ground (1 *g*) controls. The lymphocytes exhibited increases in apoptosis induced by simulated microgravity created by an RPM (Maccarrone et al., 2003). Programmed cell death in lymphocytes is caused by a mechanism based on calcium-dependent 5-LOX activation, damage of the mitochondrial membrane, the release of cytochrome c and caspase activation (Maccarrone et al., 2003). Nine years later, Battista et al. (2012) confirmed the results with the help of the microgravity simulator ROALD experiment in real microgravity on the ISS as part of the BIO-4 mission of the ESA.

On the other hand, there are also some studies reporting about reduced or inhibition of apoptotic cell death in human lymphocytes cultured in a modeled microgravity condition where the RWV culture system was used (Risin and Pellis, 2001). Another research group demonstrated a reduced expression of cell-cycle genes and downregulation of pro-apoptotic genes in lymphocytes exposed to the RWV, where authors suggested that extended exposure to simulated microgravity may result in a reduction of the cells' ability to undergo apoptosis (Kumari et al., 2009).

Even though the number of studies is limited, these findings provided a molecular background for the regulated function of lymphocytes under altered microgravity conditions.

### Macrophages

Phagocytosing of apoptotic cells by macrophages is a major key phenomenon related to active tissue restoration from wound inflammation. Macrophages constantly monitor the skin microenvironment for signals that indicate cell stress, tissue injury or infection (Murray and Wynn, 2011).

Shi et al. (2020) demonstrated that both spaceflight and simulated microgravity significantly reduced macrophage differentiation, decreased macrophage quantity and functional polarization. They also demonstrated that microgravity upregulated the expression of p53 via decreasing the expression of Mdm2 and increased p53 expression reduced apoptosis, enhanced DNA repair, and DNA damage prevention.

Yang et al. (2019) mentioned that unlike lymphocytes, U937 cells (a human macrophage cell line) cultured under simulated microgravity did not undergo apoptosis (Maccarrone et al., 2003; Maier, 2006), where lack of 5-LOX expression might protect U937 cells from apoptosis under microgravity (Maccarrone et al., 2003). In addition, the major stress protein Hsp70 (the 70 kDa heat shock protein) was shown to be up-regulated in U937 cells under simulated microgravity, which could also protect the cells from apoptosis (Maier, 2006). Furthermore, the ICAM-1 expression in the macrophage-like differentiated human U937 cells was up-regulated by simulated microgravity (Paulsen et al., 2015). The impaired PKC signaling under real microgravity (Hatton et al., 1999, 2002) might also contribute to the decreased cell motility.

An international group of researchers explored the rate at which rat macrophages adapt to weightless environments in the ISS's BioLab and reported that the production of ROS in NR8383 rat alveolar macrophages was shown to be significantly reduced by changes in real microgravity (Thiel et al., 2017). Similar results in parabolic flight and 2D clinostats showed significantly diminished ROS production during the oxidative burst in macrophages upon zymosan, curdlan, and lipopolysaccharide stimulation (Brungs et al., 2015). In another study stimulation of mouse macrophages with LPS and exposure to simulated microgravity via Rotary Cell Culture System (RCCS)-1 for 24 h resulted in significant depression TNF-α expression (Wang et al., 2014), which might lead to tumor necrosis factor-related apoptosis. Collectively, these findings provide evidence that microphage apoptosis is influenced by altered gravity conditions.

### Microvascular Endothelial Cells

The endothelium is probably one of the tissues most affected by gravitational changes regulating basic homeostatic responses such as vascular tone, angiogenesis and inflammation (Davies, 2009; Bryan et al., 2014; De Cesari et al., 2020). Endothelial cells (ECs) are known to react to microgravity by changing their proliferation rate, nitric oxide (NO) production, and cytoskeletal organization (Balsamo et al., 2014; Maier et al., 2015). The microgravity-induced impact on EC proliferation, survival, and apoptosis is probably the main causes of endothelium dysfunction and cardiovascular deconditioning that occurs in astronauts returning from space (Delp et al., 2016). Particularly microvascular ECs (HMECs) reduced proliferation or were directly induced to apoptosis when exposed to simulated microgravity (Cotrupi et al., 2005; Mariotti and Maier, 2008). In pulmonary HMECs, this behavior can be explained by increased NF-κB expression, downregulation of the PI3K/Akt pathway, and F-actin depolymerization (Kang et al., 2011). Tang et al. (2019) recently reported the involvement of miR-503-5p in the induction of apoptosis, at least in part, by inhibiting the expression of the anti-apoptotic Bcl-2 protein under simulated microgravity conditions. On the contrary, no apoptosis was observed when dermal HMECs were cultured in the RWV or on the RPM (Mariotti and Maier, 2008). This could be explained on the one hand due to HMEC heterogeneity and different experimental conditions. On the other hand, a fast induction of heat shock protein 70 (Hsp70), could protect HMECs from apoptotic stimuli by acting downstream of cytochrome c and upstream of caspase-3 (Carlsson et al., 2003; Cotrupi and Maier, 2004; Cazzaniga et al., 2019).

### Fibroblasts

**Figure 3:**
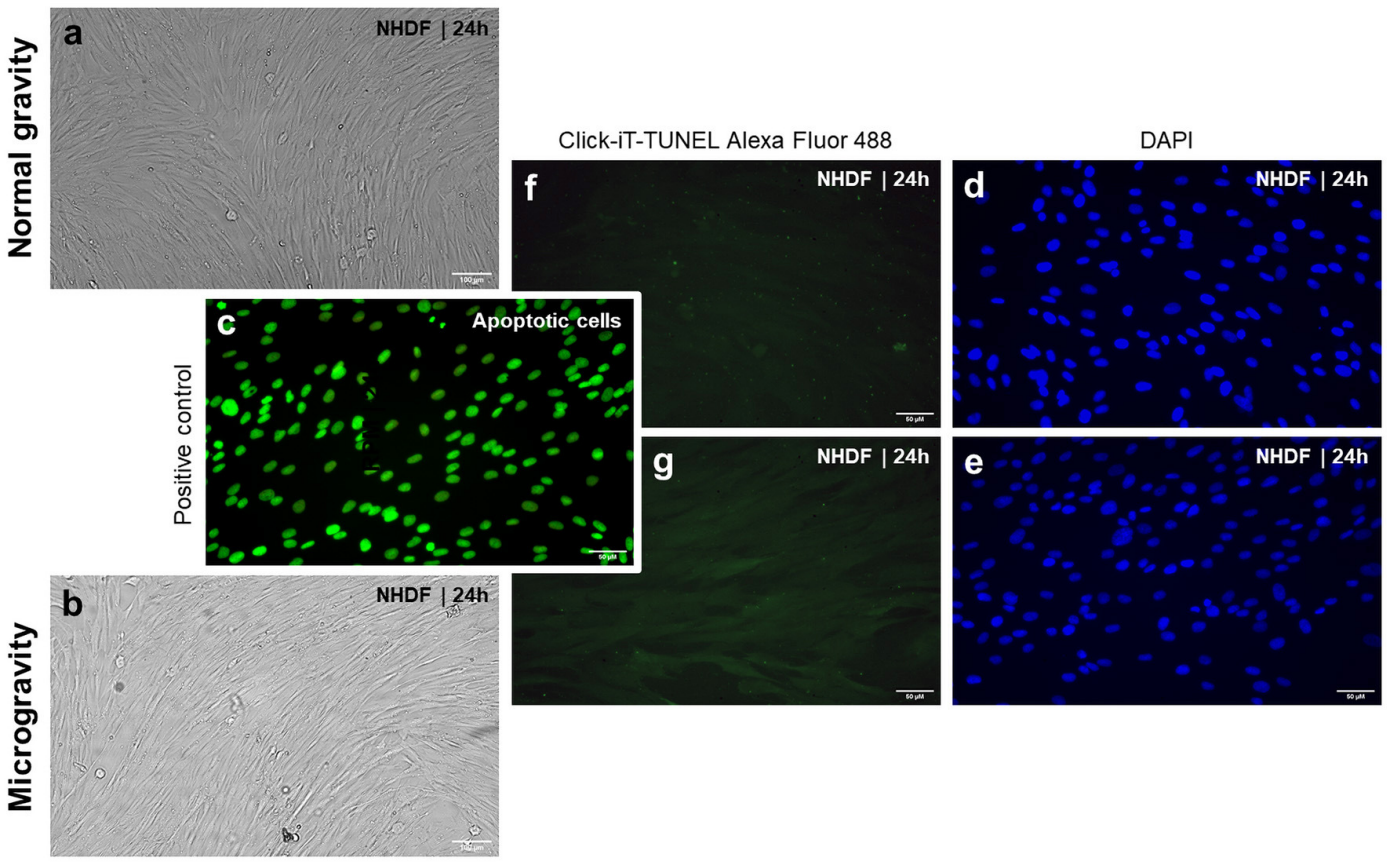
Phase contrast microscopy of normal human dermal fibroblasts (NHDF, catalog number C-12300; PromoCell GmbH, Heidelberg, Germany): **(a)** NHDF cultured under static 1 *g* conditions and **(b)** NHDF exposed to the RPM for 24 h. Click-IT terminal deoxynucleotidyl transferase dUTP nick end labeling (TUNEL) assay [(Thermo FisherScientific, Waltham, Massachusetts, USA; Click-iT TUNEL Alexa Fluor 488 (cat# C10245)] performed on NHDF exposed to 1 *g* **(f,d)**, and the random positioning machine (RPM) **(g,e)**. Green staining indicates free fluorophores in the cytoplasm in all images with the exception of the positive control **(c)**. In the positive control, samples have been pretreated with DNase to induce DNA fragmentation, which is visualized by an enrichment of the fluorophores in the nucleus. Blue staining (DAPI) highlights the cells' nuclei **(d,e)**. Green stained nuclei present apoptotic cells as shown in **(c)**. None of the applied experimental approaches (1 *g* and RPM) had induced apoptosis in the cells **(f,g)**. The evaluation was done using a Leica DM 2000 microscope equipped with an objective with a calibrated magnification of x400 and connected to an external light source, Leica EL 6000 (Leica Microsystems GmbH, Wetzlar, Germany).

ISS astronauts showed decreased skin elasticity after a 6-month space mission (Tronnier et al., 2008). Major physical stress factors responsible for this health problem are cosmic radiation and microgravity. In addition, this finding suggests possible morphological and structural alterations of the skin of humans exposed to real microgravity in space. A recent study showed that juvenile normal human dermal fibroblasts (NHDF) exposed to the RPM exhibited changes in the cytoskeleton, focal adhesion molecules, extracellular matrix, and growth (Buken et al., 2019). The RPM-exposed cells grew as adherent monolayer and as 3D aggregates revealing no dead cells. A similar result was observed previously (Beck et al., 2012). RPM-exposure and irradiation of mouse fetal fibroblasts for 24 h resulted in a decrease in apoptosis (Beck et al., 2012). Figure 3 shows the results obtained from NHDF exposed for 24 h exposed to an RPM. We used the terminal deoxynucleotidyl transferase dUTP nick end labeling (TUNEL) assay to detect DNA fragmentation by labeling the 3′- hydroxyl termini in the double-strand DNA breaks characteristic for apoptosis. The fibroblasts remained healthy and no signs of apoptosis was detectable.

Another study focused on the effects of an RPM-exposure on porcine cardiac fibroblasts which exhibited an increase in apoptosis as determined by terminal deoxynucleotidyl transferase-mediated dUTP digoxigenin nick end labeling (TUNEL) analysis, 4′, 6-diamidino-2-phenylindole (DAPI) staining, and caspase-3 detection (Ulbrich et al., 2010). Vascular Endothelial Growth factor (VEGF) and basic fibroblast growth factor (bFGF) application attenuated the number of apoptotic fibroblasts.

Fibroblasts are strongly involved in wound healing. Thus, activity changes can strongly compromise the repair process. NIH3T3 fibroblasts exposed to the RPM for 72 h showed a decrease in cell number, which might be due to apoptosis or necrosis or a decreased proliferation of these cells (Cialdai et al., 2020).

Only few data exist about the behavior of fibroblasts during spaceflight. An ISS experiment investigated possible changes in gene and microRNA (miRNA) expression profiles in confluent human fibroblast cells (Zhang et al., 2016). These were fixed after 3 and 14 d. The cell densities were greater for fibroblasts on day 14 compared with cells on day 3. Flown fibroblasts were also denser than the ground control cells on day 14. This supports the findings of Beck et al. (2012), which showed an increased cell growth of fibroblasts in space aboard the ISS. After 3 d, both the flown and ground cells were still proliferating slowly (Ki-67(+) positive cells). Moreover, gene and miRNA expression data revealed an activation of NF-κB and other growth-related pathways (Zhang et al., 2016).

Another paper reports about quiescent normal human WI-38 fibroblasts flown on the STS-93 space shuttle mission (Liu and Wang, 2008). They identified, among other differentially displayed genes, an activation of three pro-apoptotic genes and a repression of six anti-apoptotic genes. Three apoptotic genes, encoding the RNA binding protein NCL, osteopetrosis-associated transmembrane protein (*OSTM1*), and calcium- dependent phospholipid-binding protein annexin A5 (*ANXA5*) were up-regulated by spaceflight in WI-38 cells. Furthermore, six anti-apoptotic genes such as nuclear pore membrane glycoprotein (*POM121*), a small G-protein (*APMCF1*), the inhibitor of calpain calpastatin (*CAST*), a TNF receptor- associated factor binding protein (*T2BP*) a myeloid leukemia-associated SET translocation (SET), and ferritin heavy chain 1 (*FTH1*) were all downregulated (Liu and Wang, 2008). The authors suggested that the fibroblasts are triaged in space to either premature replicative senescence or apoptosis. A further study focused on fibroblasts exposed to simulated microgravity. The fibroblasts exhibited alterations in the microtubules and alpha-SMA bundles together with an impaired adherence, migration, and response to chemoattractants (Cialdai et al., 2017).

Taken together, wound healing is of enormous importance for the survival of organisms. In regard to increased space exploration adventures, we need to determine the exact influence of microgravity on dermal cells involved in the wound repair process and to research drugs with the ability to improve wound healing in space. Unfortunately, the behavior of fibroblasts in microgravity is not yet clarified. Therefore, new research projects studying human dermal fibroblasts in space have to be performed in the near future.

### Adipocytes

Adipose-derived stem cells (ADSCs) have been tested in multiple preclinical and clinical trials and have been found to enhance cutaneous wound healing through a variety of wound-healing pathways (Atala et al., 2010; Gimble et al., 2012; Shingyochi et al., 2015; Bertozzi et al., 2017). ADSCs have major potential to release angiogenic, vasculogenic, and other factors and they can stimulate their surrounding cells through the paracrine angiogenic and vasculogenic effects and accelerate wound treatment (Goodarzi et al., 2018). Nie et al. showed in their preclinical study that wound closure in normal diabetic rats can be accelerated by ADSCs, via increased epithelialization and granulation tissue deposition (Nie et al., 2011). Rigotti et al. have reported that injection of ADSCs can be effectively applied for treatment of patients with progressive wounds following radiation therapy (Rigotti et al., 2007). In cell-assisted lipotransfer (CAL) applications of ADSCs have become one of the novel stem cells transplantation strategies specifically in field of skin reconstruction (Yoshimura et al., 2008). In the CAL as an autologous tissue transfer method, fat derived ADSCs are attached to the aspirating fat which acts as living scaffolds to provide optimized condition for grafting.

The literature on ADSCs apoptosis induced by microgravity and how they behave in wound healing under altered gravity condition is very scarce. However, Ebnerasuly et al. recently demonstrated that when ADSCs were exposed to simulated microgravity by using a clinostat for 1, 3 and 7 d no significant changes in the viability or rate of apoptosis were observed. Interestingly, the research group also found increased ECM expression of *ITGB1, COL3, MMP1*, and *CD44* and declined expression of *FBN1* and *VIM* genes (Ebnerasuly et al., 2018). Hence, simulated microgravity in ADCS cells may increase their differentiation capacity toward fibroblastic cells to facilitate the wound healing process.

### Stem Cells and Wound Healing

Real microgravity during a spaceflight changes the migration behavior of stem cell-derived keratinocytes which can impair wound healing (Finkelstein et al., 2011).

Epidermal stem cells (EpSCs) play an important role in the renewal and repair of the epidermis, and are considered to have the ability to divide and differentiate into different cell types. Li and team (Li et al., 2020) have demonstrated that when EpSCs are cultured on a rotary cell culture system (RCCS) for 3 d, the amino acid metabolism pathway, lipid metabolism pathway, membrane transport pathway, cell growth and death pathways were changed by the influence of simulated microgravity.

Blaber and coworkers (Blaber et al., 2015) have studied the influence of microgravity (STS-131 STL spaceflight) on early lineage commitment in mouse embryonic stem cells (mESCs). Exposure to real microgravity for 15 d inhibited the mESC differentiation and expression of terminal germ layer lineage markers in embryoid bodies. Mechanical unloading of cells and tissues during a spaceflight revealed an inhibition of the proliferation and differentiation potential of stem cells.

Another group had shown that mesenchymal stem cells (MSCs) exposed to simulated microgravity for 72 h underwent adipogenic differentiation (Xue et al., 2017). Ratushnyy and team had found that conditioned medium from RPM-exposed adipose-derived MSCs stimulated the formation of a vessel network *in vivo*, an endothelial cell (EC) capillary-like network, and non-directed EC migration *in vitro* where an elevated expression of angiogenic regulators serpin E1, serpin F1, IGFBP, VEGF, and IL-8 was detected (Ratushnyy et al., 2018).

Beneficial effects of adipose-derived stem cells on wound healing have been reported. A further study focused on the influence of simulated microgravity using a clinostat on the gene expression of extracellular matrix (ECM) proteins and adhesion molecules in human ADSCs (Ebnerasuly et al., 2018). There were no significant changes in the cell viability detectable when the cells were exposed to a clinostat. An increase in ECM components was found and is known as one of the fibroblast markers. The authors suggest that pretreatment of adipose-derived stem cells by clinorotation may increase their differentiation capacity toward a fibroblastic phenotype (Ebnerasuly et al., 2018).

A recent study reported that it is safe to culture MSCs on the ISS. These ISS-grown MSCs exhibited an MSC-characteristic morphology and phenotype, a normal proliferative and differentiation potential. In addition, these cells were more potent in their immunosuppressive abilities compared to ground control MSCs (Huang et al., 2020). This data shows that MSCs grown in space might be useful for possible future clinical applications. It is necessary to increase the number of studies in this field to validate these results.

## Wound Healing in Space

Soon, increased commercial spaceflights and new space exploration programs to, among others, Moon and Mars will expand the amount and duration of space missions, the number of humans in space together with on board activities and elevated extravehicular work. These activities will increase the possibility to be injured and will heighten the amount of emergency surgeries in orbit. Therefore, to improve wound healing in space is currently a hot topic.

Drudi et al. reported in 2012 about the “state of the art” in-space surgery including wound healing and sutures in space (Drudi et al., 2012). As already reviewed and discussed earlier, wound healing is a well-known long-term process starting with the injury, together with tissue damage and can last months. Effective wound repair ensures the integrity of the human body and our survival.

A journey to the Mars comprises about 50 million km and thus, a cargo resupply from Earth is not possible (Thirsk, 2020). Therefore, it is mandatory to organize the onboard facilities and supplies for wound surgery in case of accidents, burns or injuries of the space crew from a diagnostic and therapeutic point of view. Additionally, long-term exposure of space travelers to cosmic radiation can negatively influence wound repair and result in degenerative tissue diseases (Thirsk, 2020).

Onboard the spacecraft medical resources such as laboratory analysis equipment, imaging, surgery, and emergency care is mandatory (Thirsk, 2020). It is of fundamental importance, that a well-trained physician with experience in remote medicine is on board as crew member, due to the communication lag it is currently not possible to guide the medical doctor remotely. In respect to the planned Mars mission, training countermeasures need to be developed in the near future. Astronaut training courses to manage in-flight accidents on long-term missions are absolutely necessary (Robertson et al., 2020).

A study investigated wound healing and mucosal immunity during a short MARS analog mission and found that stress can have significant consequences for wound healing (Rai et al., 2012). The authors concluded that the effects of stress on wound repair can influence the recovery from surgery (Rai et al., 2012).

In general, little is known about wound healing in space, because stays on the ISS or in the past on Space Shuttles and MIR were not problematic for wound repair and could be easily managed.

Interestingly, endothelial cell culture experiments performed in real microgravity revealed alterations in endothelial cell function, changes in ECM production, and 3D growth (Versari et al., 2013; Pietsch et al., 2017; Krüger et al., 2019).

Activated T cells investigated in space on board the ISS showed an activation of genes (cell cycle check-point, oxidative stress response, heat shock proteins) or by repressing genes involved in antigen recognition (Pippia et al., 2011). Battista et al. demonstrated 5-Lipoxygenase-dependent programmed cell death of human lymphocytes in space onboard the ISS (ROALD experiment) (Battista et al., 2012). This space experiment confirmed earlier data obtained with the RPM (Maccarrone et al., 2003).

Astronauts staying for a prolonged time in space exhibit an impaired immune function. The development of acquired immunity and immune responses are disturbed (Akiyama et al., 2020). Acquired immune responses are also influenced by gravitational fluctuation, as well as stressors together with cosmic radiation (Akiyama et al., 2020). A long-term spaceflight triggered a sustained stress-dependent release of endocannabinoids. Stress is stimulating inflammation-related diseases in people at risk (Buchheim et al., 2019). These health problems are resembling some features of systemic diseases which can impair wound healing on Earth. Stress, diabetes and others can negatively influence the body's response to injury and wound healing. Therefore, it is important to increase our knowledge in this field, and to design studies focusing on wound healing in space.

**Figure 4:**
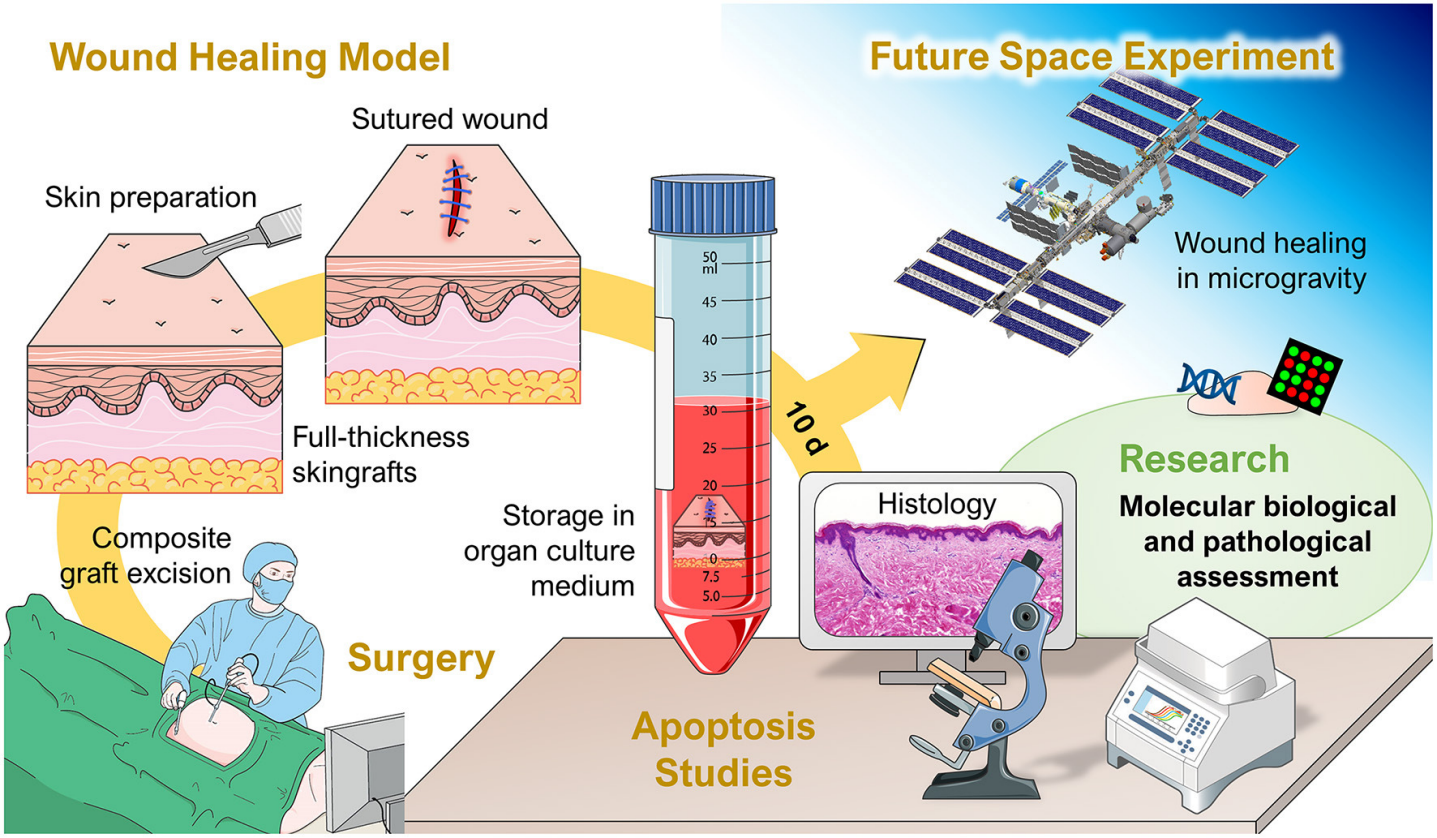
Overview of the project “Wound Healing and Sutures in Unloading Conditions.” Parts of the figure are drawn using pictures from Servier Medical Art (https://smart.servier.com), licensed under a Creative Commons Attribution 3.0 Unported License (https://creativecommons.org/licenses/by/3.0).

The international experiment of principal investigator Professor Monica Monici and co-investigators entitled “Wound Healing and Sutures in Unloading Conditions,” selected by ESA (ILSRA-2014-0043), has the objective to investigate the behavior of *in vitro* sutured wound models in real microgravity on the ISS (Figure 4). Preparatory investigations and a detailed characterization of the wound healing model had been published earlier (Riwaldt et al., 2017). Importantly, no signs of apoptosis were found in the skin samples after different culture conditions and culture duration times. The findings of the project ILSRA-2014-0043 are expected to increase our knowledge on wound healing and sutures in space. The project will help to develop novel strategies for tissue healing and management of defective healing in space and on Earth. The performed techniques and newly defined analytical protocols demonstrated to be applicable for post-flight studies on skin samples after return of the future space mission (Riwaldt et al., 2017). The principal aim of this project is to enlarge our current knowledge in wound healing in space leading to novel strategies for wound repair, tissue regeneration and engineering not only for humans in space but also for patients on Earth.

## Wound Healing and Apoptosis in Space

Wound healing is a continuous process over an extended stretch of time, a complicated and multifaceted repair procedure involving various changing cell types throughout the progression of the process. To recap from chapter 2.3, wound healing is categorized into four phases: the homeostasis and coagulation phase starts immediately after injury, followed promptly by the inflammation phase. Thereafter, the migration and proliferation phase sets in until lastly, new skin is developed during the remodeling phase (Hunt et al., 2000; Guo and Dipietro, 2010; Reinke and Sorg, 2012).

During the first phase, the wound healing cascades are instigated, with vascular constriction and fibrin clotting to stop bleeding and form a provisional wound matrix triggered by platelets, with support from leukocytes, keratinocytes, fibroblasts and endothelial cells. During this process, platelets and leukocytes deposit pro-inflammatory cytokines and growth factors (Reinke and Sorg, 2012).

While these pro-inflammatory factors attract immune cells as neutrophils and macrophages, growth factors TGF-β1, platelet derived growth factor (PDGF), fibroblast growth factor (FGF) and epidermal growth factor (EGF) stimulate tissue growth (Gosain and DiPietro, 2004; Broughton et al., 2006). Neutrophils amplify the inflammatory response, degrade necrotic cells and prevent bacterial infection (Eming et al., 2007). Macrophages are responsible for the host defense by clearing the site of pathogens and apoptotic cells within the wound, additionally they contribute to the initiation of proliferation and angiogenesis (Mosser and Edwards, 2008; Koh and DiPietro, 2011).

The most prominent cell types during the proliferative phase are fibroblasts and epithelial cells. Within the wound, fibroblasts immigrate along the fibrin network and produce extracellular matrix components like collagen, proteoglycans, glycosamyloglycans, to name a few, and so promote vascularization and filling of the wound cavity (DiPietro and Polverini, 1993; Gosain and DiPietro, 2004). The formation of new vessel in the wound filling is a complex order of events, initiated by growth factors like VEGF, PDGF, and then serine protease thrombin. These factors activate the endothelial cells of existing vessels and initiate the re-vascularization via sprouting (Reinke and Sorg, 2012).

During the last process of wound healing, the proliferation and remodeling phase, a flurry of various cell types are recruited to the close the wound. The main drivers of this stage are fibroblasts who produce the majority of materials necessary such as e.g., collagen end extracellular matrix components (hyaluronic acid, proteoglycans, glycosaminoglycans, fibronectin). In this way they form the scaffolding for cell adhesion during the tissue repair which organizes the growth and movement of the involved cells (Eckes et al., 2010; Barker, 2011). However, it should be mentioned that during the transition between the different repair phases and in order to finalize the process, cells from previous stages that are not required any longer are consistently eliminated via apoptosis throughout the entire process (Greenhalgh, 1998; Cialdai et al., 2017). An investigation in preparation to wound healing experiments during spaceflight revealed that in skin tissue cultures the healing process did not result in excessive apoptosis or necrosis after 10 d of incubation under 1 *g* conditions (Riwaldt et al., 2017).

The space environment subjects the human organism to drastic gravitational changes compared to terrestrial conditions. The lack of mechanical forces in microgravity settings causes widespread impact on the human body. Spaceflight instigates not only muscle and bone loss, immune system impairment and cardiac problems, but also negatively affects normal regenerative tissue repair and wound healing (Blaber et al., 2014; Paulsen et al., 2015; Cialdai et al., 2017). The underlying physiological changes can be described on an organic level as well as changes in protein and gene expression in the respective cell types (Blaber et al., 2014).

Cialdai et al. showed in detail how cell behavior adapts to simulated microgravity, particularly in would healing (Cialdai et al., 2017). Since fibroblasts are drivers of tissue regeneration, the focus on this study was on put on the changes of these cells after exposure to modeled microgravity. Due to a thorough rearrangement in the cytoskeleton, the adherence and migration ability of fibroblasts in wounds was significantly impaired. These findings are supported by the investigation of Davidson et al. who examined wound healing in rats sent in orbit. The animals exposed to real microgravity form 60% less collagen in an injury site compared to ground controls, hinting that wound healing in orbit is delayed (Davidson et al., 1999). Similarly, Infanger et al. showed that simulated microgravity resulted in an increase in ECM components and alteration of cytoskeletal structures in human EA.hy926 cells, moreover apoptosis increased after only 4 h of microgravity conditions (Infanger et al., 2006). In addition to the effects of the change in mechanical forces in microgravity, space radiation is another influencing factor. DNA damage due to ionizing radiation leads to increased DNA repair, cell cycle arrest and apoptosis in various cell types (Prasad et al., 2020a).

Under normal gravity, the deposition of ECM is mainly found during the proliferation phase of wound healing when fibroblast and endothelial cell proliferation peaks. Endothelial cells though were found to exhibit increased apoptosis during simulated as well as real microgravity conditions. After only 72 h of simulated microgravity conditions downregulation of the PI3K/Akt pathway and increased expression of NF-κB could be demonstrated, which may explain another aspect of the delay in healing (Kang et al., 2011).

In another *in vivo* experiment Fisher 344 rats were investigated to test wound healing responses in the orbiting Space Shuttle for 10 d (Davidson et al., 1999). The results of this study suggested that the spaceflight retarded the wound healing process and the response to exogenous stimuli.

## Ground-Based Wound Healing Studies Using Microgravity Simulators

This chapter reports about the current knowledge about wound healing studies using microgravity simulators like the RPM, 3D clinostat or the RCCS.

Microgravity may influence the process of wound healing and its progression both by changing the tissue response, apoptosis or the suture behavior. In the past, several publications showed that conditions of microgravity impaired and delayed wound repair. Until today the underlying mechanisms for an imperfect wound repair with scarring in space are not clear.

In chapter 6, we had summarized the current knowledge about various cell types active in wound repair but when exposed to microgravity several of them can become apoptotic which might result in a delay and an imperfect wound repair.

Human lymphocytes were activated and proliferate in space and on the 3D clinostat (Cogoli and Cogoli-Greuter, 1997). In free-floating cells (lymphocytes) the mitogenic response is depressed by 90% in microgravity. Maccarrone et al. showed that human lymphocytes exposed to a clinostat exhibited apoptosis by 5-lipoxygenase-mediated mitochondrial uncoupling and cytochrome c release (Maccarrone et al., 2003). These findings might influence the early phases of wound healing in space.

Another cell type of the dermis which is affected by microgravity are endothelial cells. There are several reports that endothelial cells exposed to simulated microgravity can become apoptotic and show upregulations of apoptosis signaling, downregulation of the PI3K/Akt pathway, increased expression of NF-κB, depolymerization of F-actin and that miR-503-5p can induce apoptosis of human pulmonary microvascular endothelial cells through, at least in part, inhibiting the expression of Bcl-2 (Morbidelli et al., 2005; Infanger et al., 2006; Kang et al., 2011; Maier et al., 2015; Xu et al., 2018; Tang et al., 2019; Zhao et al., 2021). In addition, clinorotation for 48 h activated autophagy in vascular endothelial cells (Wang et al., 2013).

Fibroblasts are key players in wound repair and are mechanosensitive cells (Gabbiani, 2003) and are able to produce extracellular matrix proteins (Buken et al., 2019). It is well-known that cells exposed to simulated microgravity created by an RPM show changes in the cytoskeleton and the extracellular matrix (Buken et al., 2019). Earlier studies (Morbidelli et al., 2005; Monici et al., 2011) showed that cells exposed to simulated microgravity reveal changes in extracellular matrix proteins and endothelial function, which might affect the edema behavior and tissue stiffness in wound healing. Fibroblasts exposed to a RCCS showed changes in the cytoskeleton, a reduced VEGF and elevation of COX2, responsible for an inflammatory response (Cialdai et al., 2017). Fibroblasts exposed to both, real and simulated microgravity revealed changes in their gene expression profile (Liu and Wang, 2008; Beck et al., 2012), thus suggesting the hypothesis that these alterations in fibroblast behavior, increased stress, inflammatory signals and altered gene expression pattern may be involved in disturbed wound healing in space. The findings are supported by Liu and Wang who studied normal human WI-38 fibroblasts flown on the STS-93 space shuttle mission. They reported altered key genes related to oxidative stress, DNA repair, and fatty acid oxidation in spaceflight samples (Liu and Wang, 2008). An important finding was the up-regulation of pro-apoptotic genes (Liu and Wang, 2008). The data show spaceflight induced changes in gene expression associated with cellular stress signaling, directing cells to either apoptotic death or premature senescence, which might impact wound repair and promote scarring.

Little is known about skin tissue samples exposed to simulated microgravity. One group cultured living skin equivalents (LSEs) in a microgravity environment (NASA-designed bioreactor) for 3 days (Doolin et al., 1999). Microgravity-exposed LSEs showed nuclear and cellular hypertrophy. Finally, a study of three mice living for 91 days on the ISS reported about abnormalities in the animals' skin. The spaceflight induced skin atrophy, deregulation of their hair follicle cycle, and markedly affected the transcriptomic repertoire of the cutaneous striated muscle panniculus carnosus (Neutelings et al., 2015).

The rat HS model of microgravity mimics physical inactivity by removing weight-bearing loads from the hindlimbs and producing a systemic cephalic fluid shift. Using this *in vivo* microgravity model Radek and team (Radek et al., 2008) demonstrated a delay in wound healing and wound closure in cutaneous tissue with retarded epithelial cell migration across the wound bed in the tail-suspended hindlimb-unloaded rats. The authors suggested an impaired keratinocyte and endothelial cell function during the wound healing process under simulated microgravity in the rat (Radek et al., 2008).

## New Therapies to Improve Wound Repair in Space

The principal goal is to develop novel strategies (including drugs and biochemical factors, devices, protocols and techniques) which allow a long-term stay in orbit or on other planets. This approach should promote wound repair and tissue regeneration in space. Furthermore, these strategies should be transferable to clinical applications for regenerative medicine and tissue engineering.

In this chapter, we focus on drugs and growth factors as well as biofabrication to improve wound repair.

Examples for countermeasures toward a defective neoangiogenesis are proangiogenic factors like VEGF, PDGF, and other endothelial protectives which can favor healing by modulating the neoangiogenesis process (Öhnstedt et al., 2019). Several papers reported about a reduction of VEGF in skin cells exposed to microgravity. Application of VEGF164 in rat wound healing models on Earth has demonstrated cell-protective beneficial effects on reendothelialization and vascular healing after microsurgery (Infanger et al., 2005). In addition, VEGF164 application to endothelial cells exposed to modeled microgravity exerted anti-apoptotic and cell-protective on the cells (Infanger et al., 2007).

Therefore, VEGF seems to be a promising candidate to be tested in skin wound healing models. Another option to be tested is PDGF which is approved by the FDA for non-healing wounds. Becaplerim, a gel containing recombinant PDGF (the BB isoform) is a treatment availability. Furthermore, granulocyte macrophage colony stimulating factor (GM-CSF) as injection, cream or gel can be tested.

Another growth factor with beneficial effects is epidermal growth factor (EGF). rhEGF is available as a spray and was tested in a phase III double-blind, randomized, placebo-controlled trial (Park et al., 2018). As published by Park et al. “*167 adult patients with chronic diabetic foot ulcers at six medical centers were randomized to receive routine wound care plus either topical spray treatment with 0.005% rhEGF (n* = *82) or an equivalent volume of saline spray (n* = *85) twice a day until ulcer healing or for up to 12 weeks*.” The study showed that application of spray-applied rhEGF in the patients induced a faster healing velocity and higher complete healing rate regardless of HbA1c levels (Park et al., 2018).

Recently, Cialdai et al. studied the influence of gravitational unloading on wound healing and the effectiveness of platelet rich plasma (PRP) as a countermeasure (Cialdai et al., 2020).

They used a new *in vivo* sutured wound healing model in the leech (*Hirudo medicinalis*) to evaluate the effect of microgravity on the healing process and the effects of PRP. The authors found a healing delay and structural alterations in the repair tissue, which were prevented by PRP treatment. In addition, PRP was able to counteract the microgravity-induced impairment in fibroblast migration to the wound site. In addition, it contains various growth factors. This would explain the beneficial effects of PRP in wound healing *in vivo*. Therefore, it can be assumed that PRP is also effective to promote healing in space. In respect to PRP application in space, on the ISS, its effectiveness, stability and storage conditions in microgravity have to be evaluated. Moreover, requirements to automate direct PRP preparation during spaceflight will be evaluated.

3D bioprinting technology or biofabrication of skin using endothelial cell, dermal fibroblast, and multilayered keratinocyte layers for skin tissue are currently a hot topic (Haldar et al., 2019; Barros et al., 2020).

Furthermore, biofabrication of new tissues in space on the ISS is possible, which will help humans traveling to the Moon or Mars and prosper there for years. In collaboration with ESA, OHB System AG and Blue Horizon, a research team from Dresden Technical University has developed a 3D bioprinting method for use in space, creating new skin and bone tissue from resources that might be available to astronauts, taikonauts and cosmonauts as well as space tourists traveling to the outer universe (https://www.esa.int/Enabling_Support/Space_Engineering_Technology/Upside-down_3D-printed_skin_and_bone_for_humans_to_Mars).

Taken together, several therapeutic strategies are available for humans in space on a deep exploration mission.

## Conclusions and Outlook

In the near future, increased commercial spaceflights and space exploration programs will elevate the number of space missions. With the Artemis program, NASA will land the first woman and next man on the Moon in 2024. In addition, Russia and PR China will build a Moon station together. It is envisioned as “*a complex of experimental research facilities created on the surface and/or in the orbit of the moon*.” Innovative technologies for the exploration of the lunar surface will start. Afterwards, the next goal is to send astronauts to Mars.

These new activities also mean an increased number of crew members and space tourists in space and on the Moon, which is accompanied by a higher risk of traumatic events or unexpected emergencies.

In case of emergency, ISS crew members will be transported to Earth, which is not possible in outer space. There is a continuous resupply with resources and medical equipment to the ISS. In outer space, guidance of the crew members remotely is not possible. Therefore, in this respect the spacecraft has to be self-sufficient. The crew members must be capable to manage severe situations like emergency surgery, acute trauma, wounds and burns. For these reasons, the spacecraft, space stations, Moon or Mars bases should host cutting-edge medical technology like 3D bioprinting equipment, regenerative medicine capacities, surgical instruments, functioning laboratories as well as amongst other medical equipment, freezers for blood samples, cells and tissues.

An important aspect is the behavior of the wound and/or suture and its healing. In the microgravity environment in space on the ISS are wound/suture behavior and repair processes completely unknown. Apoptosis plays an important role in normal wound healing without pathologic scarring. Several cells involved in wound repair have demonstrated an increase in apoptosis, when exposed to simulated or real microgravity. Especially lymphocytes and endothelial cells cultured in space and on microgravity simulators exhibited an increased number of apoptotic cells (Maccarrone et al., 2003; Infanger et al., 2006). In contrast, dermal fibroblasts exposed to the RPM did not reveal increases in programmed cell death (Buken et al., 2019), which implicates that mainly the early phases of wound repair might be influenced by microgravity.

*In vitro* experiments focusing on processes involved in wound repair demonstrated abnormal migration behavior, collagen formation, enhanced inflammation and reduced cellular organization (Morbidelli et al., 2005; Monici et al., 2006; Cialdai et al., 2017). Cells exposed to real microgravity revealed changes in the gene expression pattern with pro-apoptotic signaling (Liu and Wang, 2008). Mice flown for 91 d in space exhibited signs of skin senescence with skin atrophy (Neutelings et al., 2015).

Taken these findings together, it can be assumed that wound healing in space might be delayed and defective, which needs to be proven. Therefore, space missions to the ISS with focus on wound healing models are necessary.

The spaceflight proposal to the ISS with the number ILSRA-2014-0043 (PI Professor Monica Monici, Florence, Italy) with the title “Wound healing and sutures in unloading conditions” was selected by ESA. The spaceflight experiment focuses on the influence of gravity on the behavior of sutures and wound healing on the ISS. It will clarify how suturing materials and techniques can be adapted in space and it will develop strategies to improve wound healing in space. Furthermore, the project aims to improve suturing methods and wound healing avoiding scarring on Earth.

## Author Contributions

TC compiled the Table 1 and Figure 1. DM performed the NHDF RPM experiment and the TUNEL assay. MK provided Figures 2–4. All authors reviewed and evaluated the literature, wrote the article, contributed to the article, and approved the submitted version.

## Conflict of Interest

The authors declare that the research was conducted in the absence of any commercial or financial relationships that could be construed as a potential conflict of interest.
